# Synthesis of RNA-based gene regulatory devices for redirecting cellular signaling events mediated by p53

**DOI:** 10.7150/thno.55856

**Published:** 2021-03-04

**Authors:** Xinbo Huang, Mingxia Wang, Yuchen Liu, Yaoting Gui

**Affiliations:** 1 Guangdong and Shenzhen Key Laboratory of Male Reproductive Medicine and Genetics, Institute of Urology, Peking University Shenzhen Hospital, Shenzhen-Peking University-the Hong Kong University of Science and Technology Medical Center, Shenzhen 518000, China.; 2 Institute of Translational Medicine, Shenzhen Second People's Hospital, The First Affiliated Hospital of Shenzhen University, Shenzhen 518039, China.

**Keywords:** synthetic biology, gene circuit, p53 aptazyme, Cre/loxP, CRISPR.

## Abstract

**Rationale:** The *p53* gene is a well-known tumor suppressor, and its mutation often contributes to the occurrence and development of tumors. Due to the diversity and complexity of p53 mutations, there is still no effective p53 gene therapy. In this study, we designed and constructed an aptazyme switch that could effectively sense cellular wild-type p53 protein and regulate downstream gene function flexibly. The application of this artificial device in combination with Cre-LoxP and dCas9-VP64 tools achieved a precisely targeted killing effect on tumor cells.

**Methods:** The affinity of the aptamer to p53 protein was verified by SPR. p53 aptazyme and gene circuits were chemically synthesized. The function of the gene circuit was detected by cell proliferation assay, apoptosis assay and Western blot. The nude mouse transplantation tumor experiment was used to evaluate the inhibitory effect of gene circuits on tumor cells *in vivo*.

**Results:** The results of the SPR experiment showed that the p53 aptamer RNA sequence had a robust binding effect with p53 protein. The p53 aptazyme could efficiently sense wild-type p53 protein and initiate self-cleavage in cells. The Cre-p53 aptazyme gene circuit and dCas9-VP64/sgRNA mediated gene circuit designed based on p53 aptazyme significantly inhibited the growth and promoted the apoptosis of wild-type p53-deficient cancer cells *in vitro*. In addition, the gene circuits also had a significant inhibitory effect on tumors* in vivo*.

**Conclusion:** The study developed a novel and efficient ribozyme switch for p53-specific recognition and provided a modular strategy for aptazyme binding to cellular proteins. In addition, the p53 aptazyme successfully inhibited tumor growth through a combined application with other synthetic biological tools, providing a new perspective for cancer therapy.

## Introduction

Synthetic biology has been applied in several fields, and the construction of controllable and sensitive gene circuits is an important means to achieve simulated regulation of cellular physiological activities 1, 2. The ribozyme switch is an artificial gene regulatory device developed in recent years that is composed of three modules: a sensor (detecting user-defined input), a linker (transmitting information), and an actuator (activating target gene expression) 3. After binding to the ligand, the ribozyme changes its conformation and induces the expression of downstream reporter genes, such as fluorescence signals 4. This switch improves the previous protein-responsive RNA devices by achieving better controllability of gene expression, structure assembly, and target gene specificity 5-7.

A common ribozyme switch consists of a hammerhead ribozyme and an aptamer, which is also called “aptazyme” 8-10. Hammerhead ribozyme is an RNA motif with a specific wishbone-shaped structure characterized by its small size and stability 11. As a *cis*-acting element, the aptazyme can regulate ligand-mediated cleavage without the aid of protein 12. Based on its flexibility, rapid response and simple regulation mode, the aptazyme has been applied in the development of *in vivo* sensors, gene therapy, and biological processors 9, 13, 14. However, the existing aptazymes are mainly applied to microbial cells instead of mammalian cells 15-17. In addition, the majority of existing aptazymes were reported to respond to small-molecule ligands, and few of them interacted with cellular proteins and performed desired functions 18. These limitations are worth further investigation to achieve a future applicable tool.

The* p53* gene is a key tumor suppressor gene with the highest mutation rates in cancer 19. Mutations in p53 have been shown to abrogate its tumor suppressor function. Recent studies have additionally reported that mutated p53 directly participates in tumor development and functions as an oncogene 20-22. Various treatments targeting p53 mutations have been investigated intensively, including mutant p53 protein inhibitors and transduction of wild-type p53 protein via viral vectors 23-25. In synthetic biology, genetic p53 sensors to detect the state of p53 and activate cancer cell-targeted killing were developed 26, 27. They are usually designed to work independently; thus, improvements to achieve random combinations with other synthetic modules are required. An ideal sensor should display predictable function and seamless interoperability with other synthetic biology tools 28, 29. As RNA structures that can specifically bind ligands, aptazymes have the potential to be developed as sensors. Accordingly, we are committed to designing a p53 aptazyme switch that can regulate cellular function in mammalian cells.

In our previous study, we used a p53 aptamer that specifically binds to the p53 protein to construct gene circuits to direct cellular information 30, 31. In a previous study, Win et al*.* reported an extensible RNA-based framework for engineering small molecule-controlled gene-regulatory systems that exhibited tunable regulation, design modularity and target specificity 32, 33. Inspired by this study, we hypothesized that this approach could also be used to detect intracellular proteins, including p53. To test this idea, we developed a sensitive and modulatory RNA-based aptazyme that sensed the wild-type p53 protein and controlled the targeted killing of cancer cells.

The p53 aptazyme we designed has a simple structure and predictable functions. The system takes endogenous p53 protein as the input signal to regulate the activity of aptazymes. It does not need to add an exogenous inducer, which reduces the influence of exogenous inducer on cells and reduces the complexity of operation. To apply the p53 aptazyme to the tumor-specific killing gene circuits, we integrated the p53 aptazyme into the Cre-loxP and dCas9/VP64-sgRNA systems to construct two tumor-specific killing circuits that can kill tumor cells both *in vitro* and *in vivo*. The construction strategy of the p53 aptazyme provides a new approach for the synthesis of specific cancer-killing gene circuits.

## Material and methods

### Plasmid construction

To construct dual-luciferase reporter vectors, we inserted the hRluc-p53 aptazyme downstream of the SV40 promoter and hluc downstream of the HSV-TK gene. To construct the hTERT-Cre-p53 aptazyme-*late poly (A)* vector, the p53 aptazyme sequence was inserted into the 3'-UTR of Cre. The fusion *Cre-P53 aptazyme gene* was driven by the hTERT promoter. To construct the U6-sgRNA-*late poly(A)*-hTERT-dCas9-VP64-p53 aptazyme-*late poly (A)* vector, we inserted the p53 aptazyme and the negative control sequence (complete hammerhead ribozyme sequence) into the 3′-UTR of VP64. At the same time, lentivirus vectors containing the same elements were constructed by the Syngentech company (Beijing, China). Then, the lentiviral vectors and virus packaging vectors were cotransfected into 293T cells. After that, the virus solution was collected, concentrated and subjected to titer determination. The relative sequences of these vectors are presented in Supplementary Table 1. The maps of plasmids used in this article are presented in Figure S4.

### Dual luciferase reporter assay

Cells were seeded in 24-well plates (1×10^5^ cells per well) and transfected with synthetic plasmids. The p53-aptazyme sequence was inserted 3' UTR of the hRluc in the pSiCheck2 vector by Syngentech Co., Ltd (Beijing, China). These plasmids were transfected into HFF, 293T, HCT116 p53+/+, HCT116 p53+/+, 5637, G361 and SCl-1 cells using Lipofectamine 3000 Transfection Reagent. Luciferase activity was tested 48 h after transfection by the dual luciferase assay system (Promega, Madison, WI, USA) according to the manufacturer's protocol.

### Cell lines and cultures

HFF (isolated from foreskin tissue), 293T and HCT116 p53-/- cells were cultured in DMEM (Invitrogen, Carlsbad, CA, USA) with 10% fetal bovine serum (FBS), 100 µg/mL streptomycin and 100 U/mL penicillin. The 5637, G361, SCL-1, HeLa and HCT116 p53+/+ cells were cultivated in RPMI 1640 with 10% fetal bovine serum (FBS), 100 µg/mL streptomycin and 100 U/mL penicillin. All cells were cultured at 37 °C in an atmosphere of 5% CO_2_.

### Cell transfection

Cells were cultivated on plates for 24 h with Opti-medium (Invitrogen, Carlsbad, CA, USA) prior to transfection. DNA transient transfection assays were conducted using Lipofectamine 3000 (Invitrogen) according to the manufacturer's protocol. Transfection of cells was performed following growth to 70-80% confluency in 6-well plates with 2.5 μg of plasmid, followed by collection of transfected cells after at least 48 h.

### SPR

The aptamer RNA was diluted to 10 ug/ml with DEPC+PBST for binding to the SA sensor chip (Fortebio, 18-5019). The p53 protein (KMH1837, KMD Bioscience) was diluted to 100nM with DEPC+TBST. The aptamer was first immobilized on the SA sensor chip followed by the binding of 200 ul 100 nM p53 protein in the sample well. The reference well was treated the same, except for replacing the 200 ul p53 protein with 200 ul DEPC+PBST solution. The binding ability between the aptamer and p53 protein was detected using the Octet RE96E (Fortebio).

### RT-qPCR

According to the manufacturer's protocol, RNAeasy TM RNA Isolation Kit (Beyotime Biotechnology, China) was used to isolate total RNA from cells under different illumination conditions. cDNA was synthesized using BeyoRT TM II cDNA Synthesis Kit (Beyotime Biotechnology, China). The mRNA expression was determined using SYBR Green qPCR MasterMix (Takara, Dalian, China), with gapdh as the control. The relative mRNA (p53 and DT) level was calculated by △△Ct method. The primers for *gapdh*, *p53* and *DT* were shown in the following sequences, with directions ranging from 5'to 3':

*gapdh* (F): TCCCATCACCATCTTCCA

*gapdh* (R): CATCACGCCACAGTTTCC

*p53* (F): CCTCAGCATCTTATCCGAGTGG

*p53* (R): TGGATGGTGGTACAGTCAGAGC

*DT* (F): AAGGGCTTTTACAGCACCGA

*DT* (R): CCTTCTTGATGGTCTCGGCA

### Western blot

Cells were lysed by RIPA lysis buffer with protease inhibitor cocktail. Twenty micrograms of total protein were separated on sodium dodecyl sulphate-polyacrylamide gel electrophoresis (SDS-PAGE) gels and then transferred onto PVDF (polyvinylidene fluoride) membranes. The sample proteins were incubated with primary antibodies overnight at 4 °C, and then incubated with secondary antibodies for 1 h at room temperature. An ECL Chemiluminescence Detection Kit (Millipore) was used to visualize the immunoreactive proteins. The antibodies information: p53 (1:500, sc-126, Santa Cruz Biotechnology); Bax (1:1000, AF0057, Beyotime); Bcl-2 (1:1000, AF6285, Beyotime); DTA (1:1000, PAV4207, KMD Bioscience).

### Cell proliferation assay

The effects of the negative control and experimental plasmids were measured using the Cell Counting Kit (CCK8) assay. After cell counting, approximately 5 × 10^3^ cells/well were seeded in a 96-well plate and preincubated for 10-12 h. Next, 10 μL of Cell Counting Kit solution was added to each well at 0, 24, 48 and 72 h time points. After 60 min of incubation, the optical density (OD, 450 nm) value of each well was calculated using the CCK8 reader machine (Bio-Rad, Hercules, CA, USA). Experiments were repeated at least three times.

### Flow cytometry assay

Cells were seeded in 6-well plates and infected with the negative control lentivirus and the test group lentivirus. Then, the cells were harvested using trypsin without EDTA. In the Cre-p53 aptazyme experiment, the Annexin V PE/7-AAD Apoptosis Detection Kit (TransGen, Beijing, China) was used to double-stain cells with 7-AAD and PE according to the manufacturer's protocols. In the CRISPR-p53 aptazyme experiment, the FITC Annexin V Apoptosis Detection Kit (TransGen, Beijing, China) was used to double-stain cells with FITC-Annexin and PI according to the manufacturer's protocols. A flow cytometer (EPICS, XL-4, Beckman, CA, USA) was used to observe cell apoptosis. Cells were discriminated into living cells, early apoptotic cells, late apoptotic cells and dead cells. The ratio of early apoptotic cells in the negative control group to the experimental group was used as an index for comparison. The experiments were repeated at least three times.

### Xenograft tumor model

The relevant vectors were packed into lentivirus using a Lentiviral Packaging Kit (Syngentech Co, Ltd, Beijing, China) according to the manufacturer's protocol. Male immunodeficient BALB/c nude mice (5-6 weeks old) were purchased from Beijing Wei-tong Li-hua Laboratory Animals and Technology Ltd. Approximately 5 × 10^6^ G361, 5637 and HCT116 p53+/+ cells were suspended in 100 μL of Matrigel (BD Biosciences, Franklin Lakes, NJ, USA). The mixture was injected subcutaneously into the dorsal flank regions of BALB/c nude mice. After 3 weeks of observation, the mice were euthanized, and the subcutaneous weight of each tumor was determined. Tumor volumes were calculated according to the following formula: 0.5 × length × width^2^. Mice were euthanized, and the subcutaneous weight of each tumor was determined. Our experimental procedures were approved by the Institutional Ethics Review Board.

### Statistical analysis

All experimental data from at least three independent experiments are presented as the means ± standard deviation (SD). Data were analyzed using SPSS 22.0 software (SPSS Inc. Chicago, II, USA). Data obtained from the CCK8 assay were analyzed via ANOVA, and the independent samples T*-*test was applied to evaluate other data. A two-tailed value of P < 0.05 was considered statistically significant.

## Results

### Construction and verification of the p53 aptazyme

**Figure 1:**
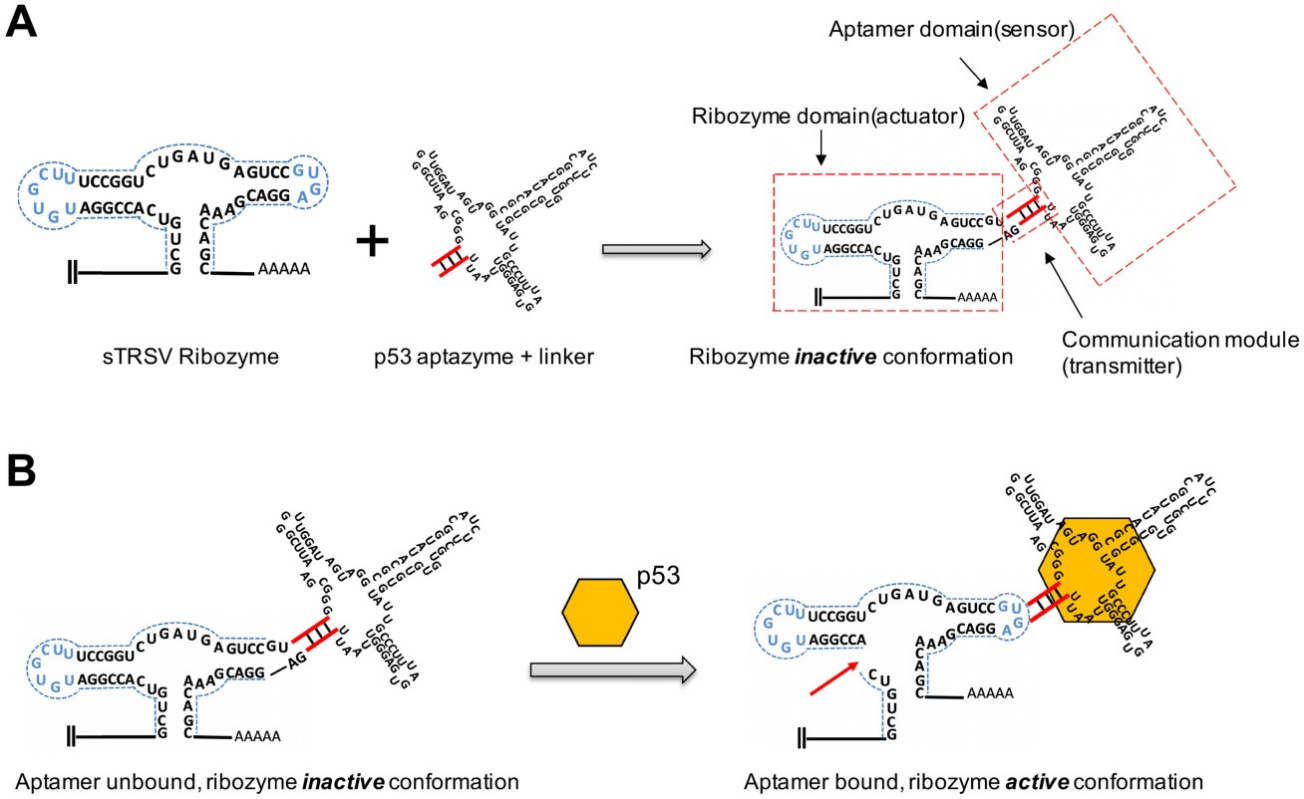
** Design strategy for p53 aptazyme switch: (A)** Aptamer ribozyme based on a hammerhead sTRSV ribozyme and a p53 aptamer. The aptazyme includes a ribozyme domain, an aptamer domain and a communication module. **(B)** In the presence and binding of p53 protein, the aptazyme experienced self-cleavage. The color scheme is as follows: blue dashed line, loop sequence; red line, linker sequence; red arrow, cleavage site; yellow hexagon, p53 protein.

To investigate whether the p53 protein could bind to aptazyme and regulate hammerhead ribozyme activity, we constructed a p53-responsive ribozyme switch (p53 aptazyme). The structure of the aptazyme is presented in Figure 1A, and the linkage of the hammerhead ribozyme, the linker sequence and the p53 aptamer are presented. In the absence of p53, the binding of the aptamer and the hammerhead sTRSV ribozyme prevented its cleavage activity. Nevertheless, with p53, the loop structure of the hammerhead ribozyme was recovered, and cleavage was activated (Figure 1B).

**Figure 2:**
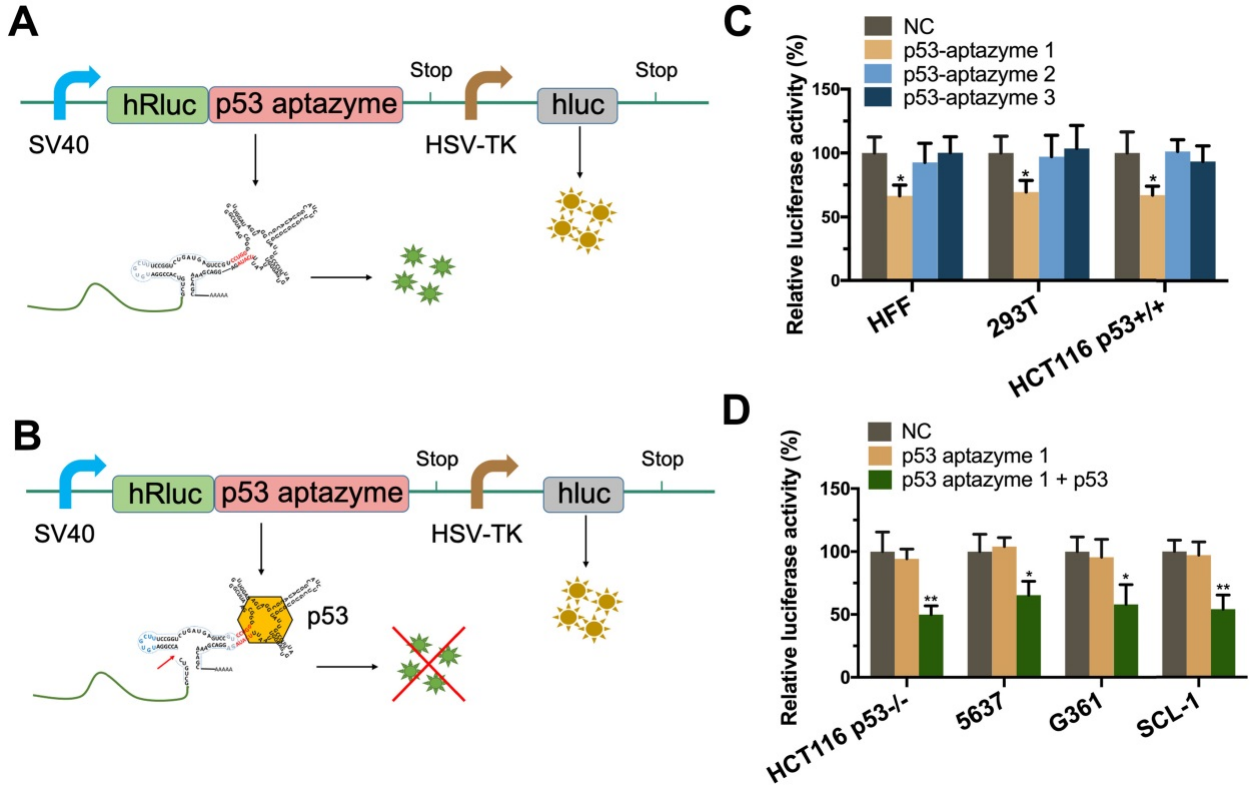
** Specificity verification of p53 aptazyme linker sequences: (A, B)** Construction of the dual luciferase plasmid, where the p53 aptazyme was inserted into the 3'UTR of the hRluc gene. The expression of p53 aptazyme was determined by relative luciferase activity (hRluc/hluc) (green/yellow). Dual fluorescence emission in the absence of p53 protein (A). Single fluorescence emission in the presence of p53 protein (B). **(C)** Effects of p53 aptazyme (linker-1, linker-2, linker-3) on the relative luciferase activity in wild-type p53 cells (HFF, 293T and HCT116 p53+/+) compared to the negative control. **(D)** Effects of p53 aptazyme linker-1 and p53 aptazyme linker-1 with p53 overexpression on the relative luciferase activity in wild-type p53-deficient cells (HCT116 p53-/-, 5637, G361 and SCL-1) compared to the negative control. Data are presented as the means ± SD from at least three biological replicates. (^*^P < 0.05, ^**^P < 0.01)

First, we tested the binding force between p53 aptamer RNA and wt-p53 protein. The results of the SPR experiment showed that the p53 aptamer RNA sequence had a robust binding effect with the protein (Figure S6). To screen effective linker sequences, we selected three sequences with good scores from a previous study and constructed corresponding p53 aptazymes (p53 aptazymes 1/2/3) 32. The three p53 aptazymes were inserted into the 3'UTR of the human Renilla luciferase gene (hRluc), with the unmodified dual luciferase plasmid vector used as the negative control (Figure 2A). Before the test, the expression of p53 protein was detected in eight cell lines (HFF, 293T, HCT116 p53+/+, HCT116 p53-/-, 5637, G361, SCL-1, Hela) (Figure S7).

Three p53 wild-type cell lines, HEK 293T (human embryonic kidney cells), HCT116 p53+/+ (human colon cancer cells) and primary cultured HFF (human foreskin fibroblast cells), were used to test whether the aptazymes could sense endogenous p53. The efficiency of the aptazymes was assessed by comparing the relative fluorescence between the experimental and control groups. Finally, we found that only the linker-1 sequence could transmit an aptamer-ligand binding signal (Figure 2C).

To investigate the ability of the synthetic aptazyme to discriminate p53 wild-type cells and p53 mutant cells, luciferase assays were performed in four cell lines, including HCT116 p53-/- (human colon cancer cells with p53 knockout), 5637 (bladder cancer cells), SCL-1 (cutaneous squamous cell carcinoma cells) and G361 (human melanoma cells). According to previous p53 mutation analysis and sequencing of the *p53* gene in 5637 and SCL-1 cells, the results showed homozygous* p53* mutation in 5637 cells and heterozygous *p53* mutation in SCL-1 cells 34.

As shown in Figure 2C, after transfection, the relative hRluc luciferase activity of the p53 aptazyme1 group was significantly reduced in HFF, 293T and HCT116 p53+/+ cells but not in p53 mutant cells compared with the negative control (NC). However, when we overexpressed wild-type p53 protein in p53 mutant cells, the relative hRluc luciferase activity was also significantly decreased (Figure 2D). These results demonstrated that the synthesized p53 aptazyme1 could efficiently sense wild-type p53 protein and initiate self-cleavage.

### Construction of the Cre-LoxP mediated gene circuit based on the p53-aptazyme device

According to the results of the dual luciferase assay, we inferred that p53 aptamer ribozyme 1 could specifically bind to p53 protein and activate the activity of hammerhead ribozyme. To produce a targeted killing effect on tumor cells, we assembled the ribozyme switch (p53 aptazyme 1) with the Cre-LoxP system, which is commonly used for genetic pathway modifications. In our design, the p53 aptamer functioned as the sensor for endogenous p53 and generated a cell-killing effect in p53-deficient cells. Two vectors were constructed: one expressing the Cre-p53 aptazyme and the other containing the LoxP-EGFP-LoxP-DT (diphtheria toxin, DT) sequence 35. The p53 aptazyme sequence was inserted into the 3′-UTR of Cre and driven by the mutant human telomerase reverse transcriptase (hTERT) promoter (Table S1) 36, while the LoxP-EGFP-LoxP-DT circuit was driven by the human elongation factor 1A promoter (hEF1a) promoter. The hTERT promoter is highly expressed in cancer cells, thus facilitating our systems' ability to discriminate a tumor microenvironment from normal tissue 36.

**Figure 3:**
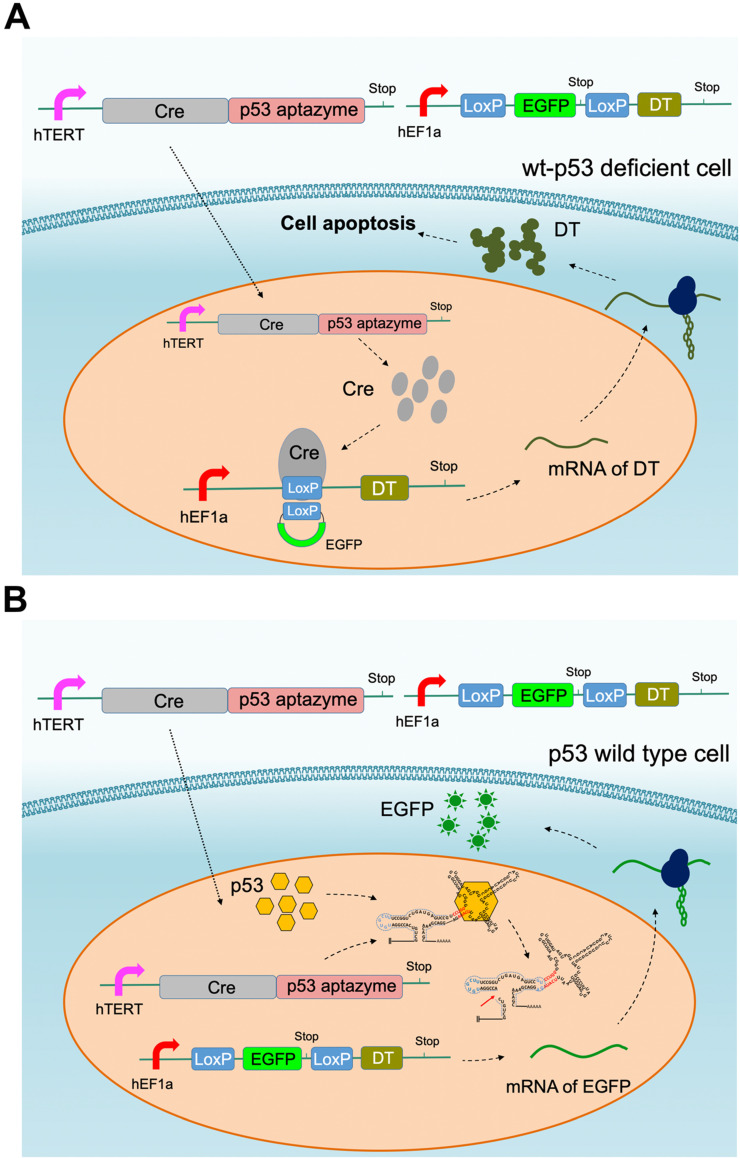
** Cre-p53 aptazyme mediated cell apoptosis: (A)** A depiction of the Cre-LoxP gene switch that included two plasmids: the Cre-p53 aptazyme driven by the hTERT promoter and the LoxP-EGFP-LoxP-DT driven by the hEF1a promoter. In wild-type p53 deficient cells, diphtheria toxin (DT) was produced and induced apoptosis. **(B)** A depiction of the switch's action in wild-type p53 cells, where the interaction between p53 and p53-aptazyme allowed the degradation of Cre mRNA and expression of EGFP.

In this gene circuit, when wild-type p53 protein bound the p53 aptamer, the ribozyme was activated, the fusion transcript Cre-ribozyme aptamer degraded and EGFP was produced (Figure 3B). In contrast, the ribozyme in p53 mutant cells remained inactive. LoxP sites were spliced, leading to the production of the diphtheria toxin downstream of EGFP (Figure 3A). Through this design, the gene circuit achieved p53 sensing and tumor cell-targeted killing.

### The Cre-p53 aptazyme gene circuit inhibited the growth and promoted the apoptosis of wild-type p53-deficient tumor cells *in vitro* and *in vivo*

**Figure 4:**
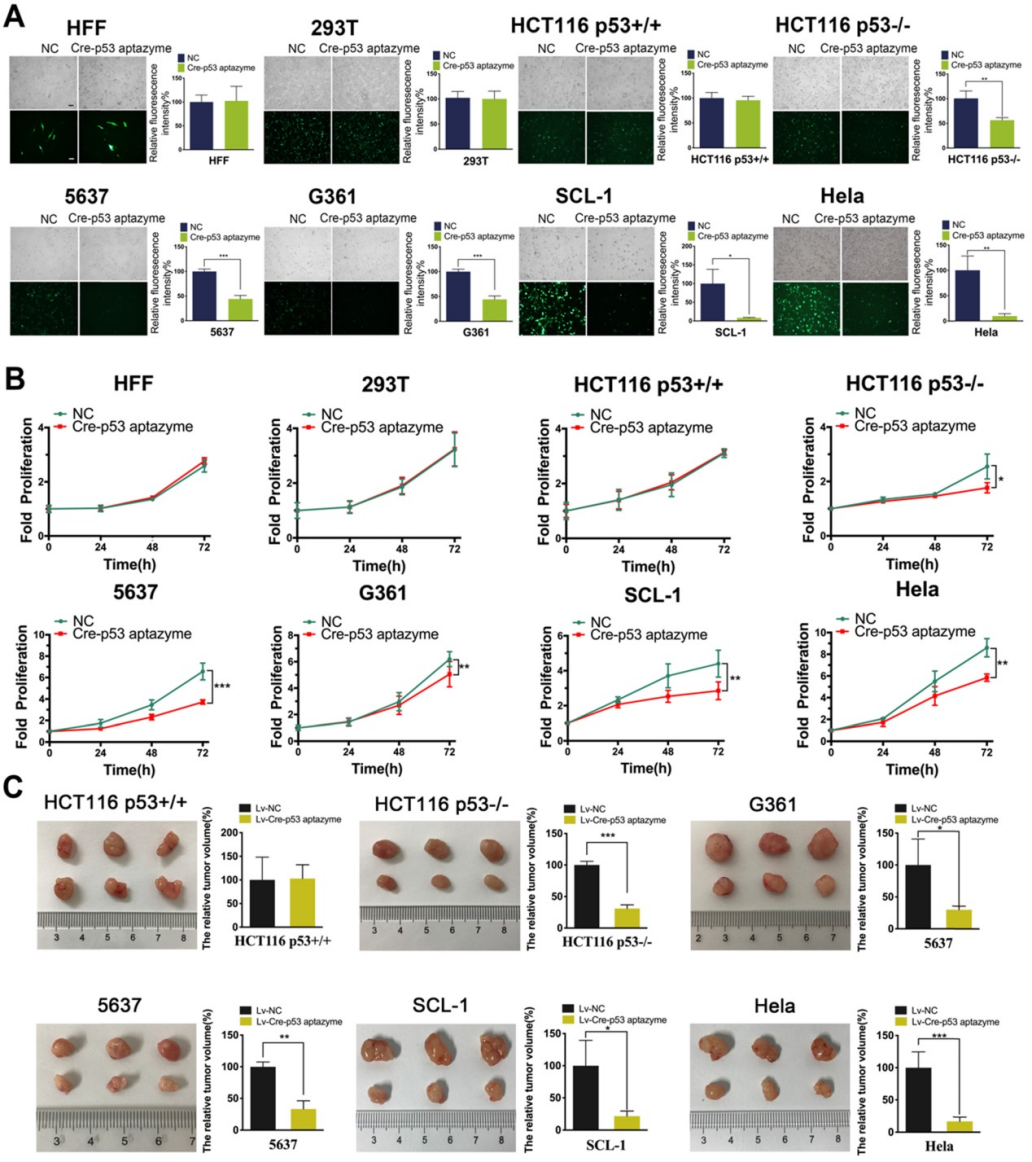
** The proliferation of wild-type p53-deficient cells was inhibited by the Cre-p53 aptazyme system *in vitro* and* in vivo*: (A)** The expression of EGFP was assessed under Cre-p53 aptazyme treatment or negative control using wild-type p53 cells (HFF, 293T and HCT116 p53+/+) and wild-type p53 deficient cells (HCT116 p53-/-, 5637, G361, SCL-1 and HeLa). **(B)** CCK-8 assay representing the effect of Cre-p53 aptazyme and the negative control on cell proliferation using wild-type p53 cells and wild-type p53 deficient cells*.* **(C)** The effect of Cre-p53 aptazyme and the negative control on tumor growth using wild-type p53 cells and wild-type p53-deficient cells*.* Data are presented as the means ± SD from at least three biological replicates. (^*^P < 0.05, ^**^P < 0.01, ^***^P < 0.001)

Furthermore, we investigated whether the Cre-p53 aptazyme gene circuit could inhibit p53-deficient tumors. As shown in Figure 4A, we observed no significant difference in relative fluorescence intensity between the Cre-p53 aptazyme and NC (Cre-Ribozyme) groups in the HFF, 293T and HCT116 p53+/+ cell lines. However, significant differences in relative fluorescence intensity between the Cre-p53 aptazyme and NC (Cre-Ribozyme) groups in the HCT116 p53-/-, G361, 5637 and SCL-1 cell lines were observed. Relative fluorescence values were calculated using ImageJ software. By detecting the expression of DT mRNA and protein in cell lines, we found that the expression of DT was increased in the above cell lines (Figure S1A) (Figure S8). Relative cell activity in the two groups was measured using the Cell Counting Kit-8 (CCK-8) assay. The proliferative activities of HFF, 293T and HCT116 p53+/+ cells in the p53 aptazyme group were not markedly different compared with those in the NC group. In contrast, the Cre-p53 aptazyme device suppressed cell growth in the p53-deficient cell lines (HCT116 p53-/-, 5637, G361, SCL-1 and HeLa) (Figure 4B). Meanwhile, the Cre-p53 aptazyme device promoted cell apoptosis of wild-type p53-deficient tumor cells (Figure S2). This conclusion was supported by the detection of apoptosis-related protein Bax/Bcl-2 (Figure S8).

The inhibitory effect of the Cre-p53 aptazyme device *in vitro* prompted further investigation of the efficacy of this strategy to regulate cell growth. Cancer cell lines were selected for tumor cell transplantation in nude mice. The Cre-p53 aptazyme system effectively reduced the tumor size in nude mice injected with p53-deficient cells but had no effects in those injected with wild-type cells (HCT116 p53+/+) (Figure 4C). The data obtained from the different assays validate the role of the p53 aptazyme device in the Cre-LoxP system, with specific recognition of p53 and induction of apoptosis in p53-deficient cells.

### Construction of the dCas9-VP64/sgRNA mediated gene circuit based on the p53-aptazyme device

**Figure 5:**
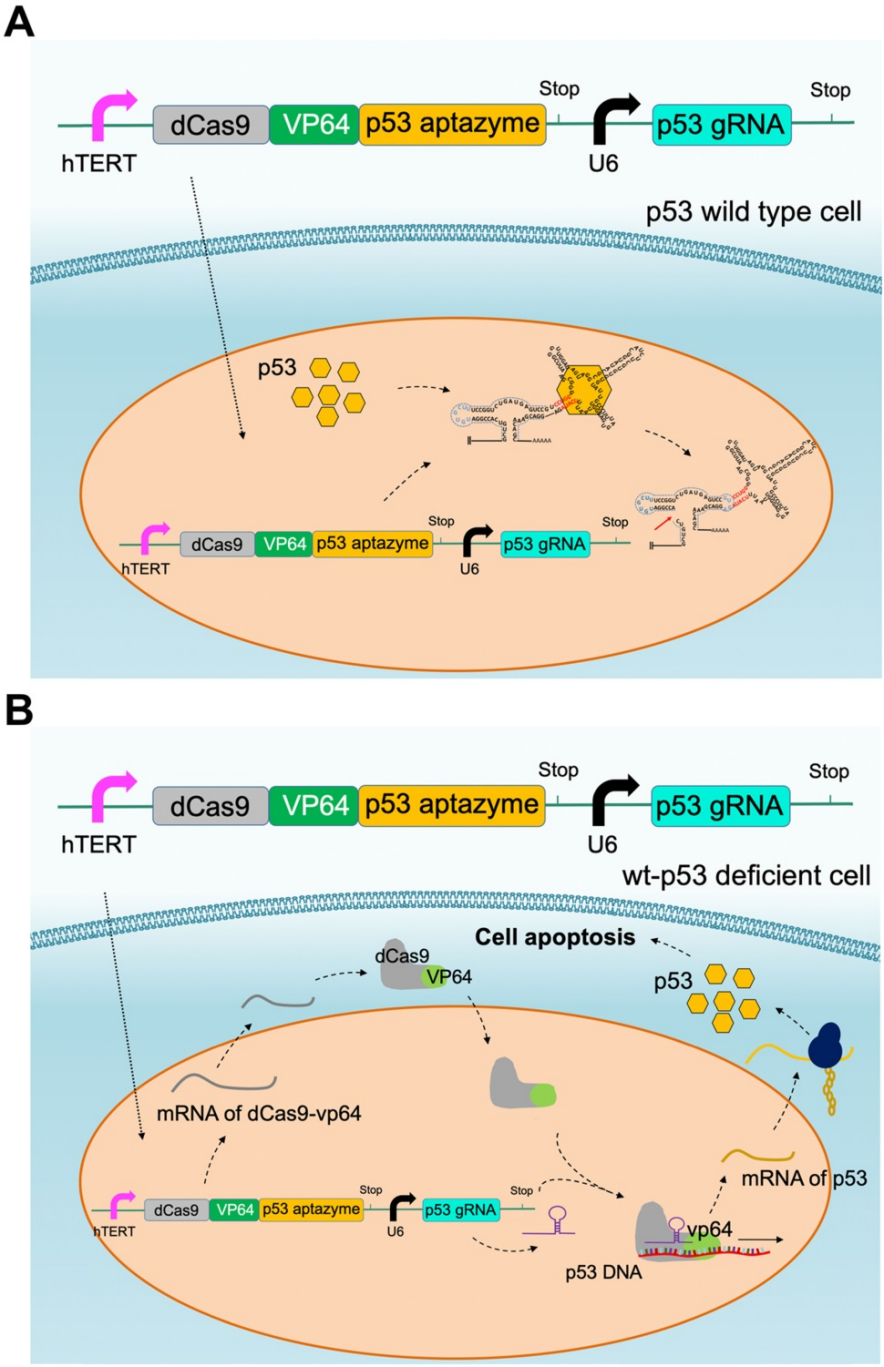
** CRISPR-p53 aptazyme mediated cell apoptosis: (A)** A depiction of the CRISPR-p53 gene switch composed of the dCas9-VP64-p53 aptazyme driven by the hTERT promoter and the p53 gRNA driven by the U6 promoter. In wild-type p53 cells, p53 prevented the production of dCas9-VP64 and halted the circuit.** (B)** A depiction of the switch's action in wild-type p53 deficient cells. dCas9-VP64 activated the expression of wild-type p53 under sgRNA (p53) guidance, which inhibited cell growth.

The simplification of the gene circuit is beneficial to the carrier delivery efficiency and system stability. The Cre aptazyme is relatively large and requires two carriers rather than a single carrier to deliver the cancer-specific killing system. On the other hand, the CRISPR system can achieve a cancer-specific killing function by constructing a vector. Here, we have developed a dCas9-VP64 tool that can effectively activate the wild-type p53 allele in cancer cells and play a role in cancer cell killing. The designed circuit expressing the CRISPR-dCas9 and p53 aptazyme was encoded on plasmids (Figure 5). The single plasmid system simplified the complexity of the vectors and showed better efficiency and adaptability. At the same time, an untargeted sgRNA control vector was designed for the sgRNA domain, and an unmodified ribozyme control vector was designed for the p53-aptazyme domain. Without exogenous DT expression, the CRISPR-p53 device could activate endogenous p53 protein in cells. In wild-type p53 cells, the ribozyme was activated, and the dCas9-VP64 fusion gene was inactivated (Figure 5A). In contrast, p53 protein was overexpressed by dCas9-VP64 in wild-type p53-deficient cells (Figure 5B). The induction of cell signals and targeted killing effects were achieved simultaneously by a single CRISPR-p53 aptazyme system (Figure S5).

### The dCas9-VP64/sgRNA-p53 aptazyme switch inhibited the growth and promoted the apoptosis of wild-type p53-deficient cells *in vitro* and *in vivo*

**Figure 6:**
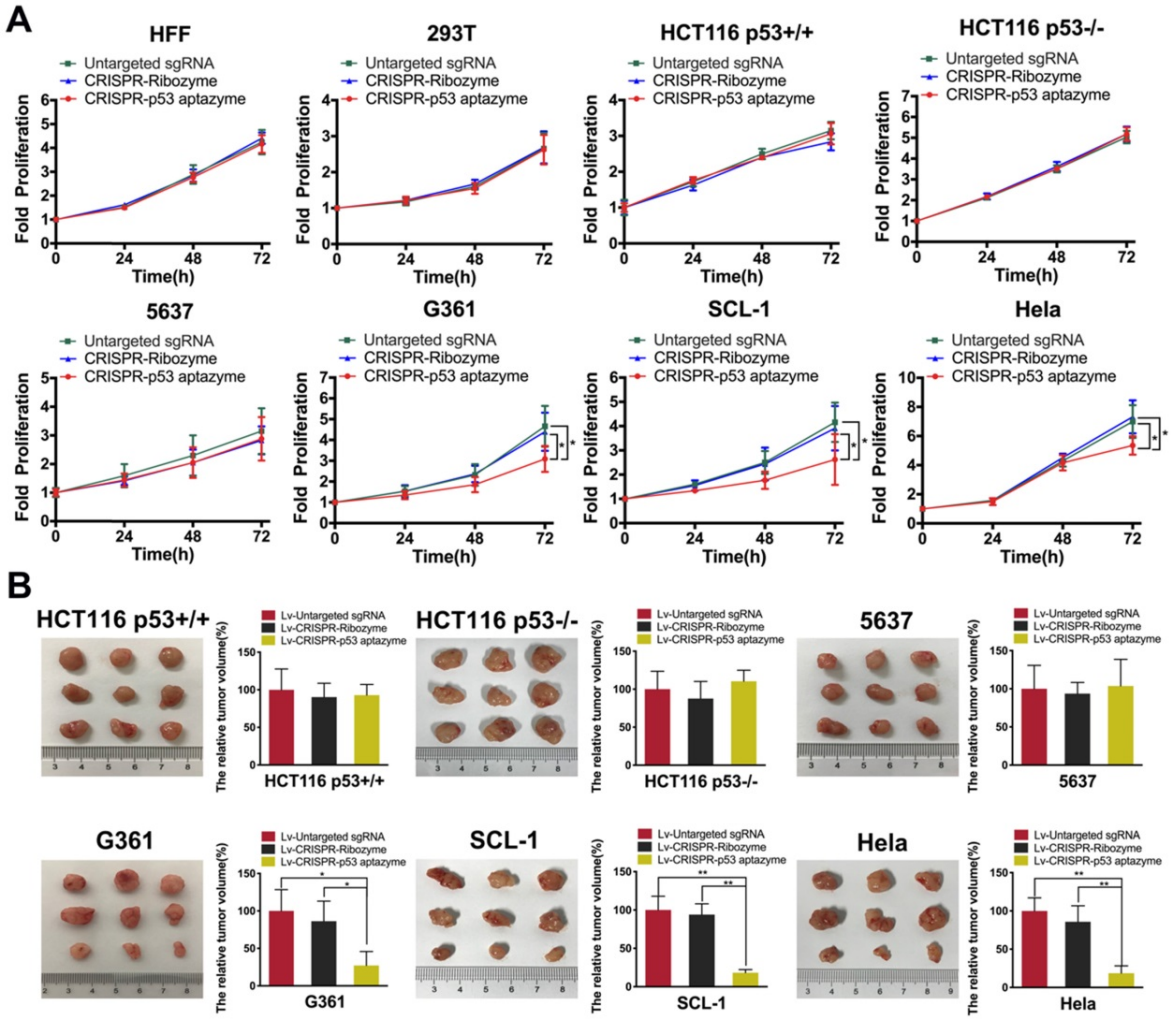
** The proliferation of several wild-type p53-deficient cells was inhibited by the CRISPR-p53 aptazyme system *in vitro* and* in vivo*: (A)** CCK-8 assay representing the effect of untargeted sgRNA, CRISPR-ribozyme and CRISPR-p53 aptazyme on cell proliferation using wild-type p53 cells (HFF, 293T, HCT116 p53+/+) and wild-type p53 deficient cells (HCT116 p53-/-, 5637, G361, SCL-1 and HeLa)*.* **(B)** The effects of untargeted sgRNA, CRISPR-ribozyme and CRISPR-p53 aptazyme on tumor growth using wild-type p53 cells and wild-type p53-deficient cells*.* Data are presented as the means ± SD from at least three biological replicates. (^*^P < 0.05, ^**^P < 0.01, ^***^P < 0.001)

To validate the activation of wild-type p53 expression, we transfected dCas9-VP64/sgRNA-p53 aptazyme plasmids into several cell lines. In CRISPR-p53 aptazyme-treated cells, we observed significantly higher expression of p53 in wild-type p53-deficient cells by quantitative real-time PCR (Figure S1B). To study whether the switch could inhibit wild-type p53-deficient tumor cells, proliferation was examined by CCK-8 assay. Cell proliferation of the p53 aptazyme group showed no difference from the negative controls in HFF, 293T, 5637, HCT116 p53+/+ and HCT116 p53-/- cells. However, in G361, SCL-1 and HeLa cells, we observed a significant difference in proliferation between the p53 aptazyme and two control groups (“Untargeted sgRNA” and “CRISPR-Ribozyme”). In G361, SCL1 and HeLa cells, wild-type p53 at the allelic locus was activated by dCas9-VP64, and cell growth was inhibited (Figure 6A). Moreover, the dCas9-VP64-p53 aptazyme system promoted cell apoptosis in G361, SCL-1 and HeLa cells (Figure S3). To some extent, the dCas9-VP64-p53 ribozyme tool could identify wild-type p53-deficient tumor cells and activate endogenous p53 to induce cell apoptosis (Figure S9). Plasmids were packaged into lentiviruses and transduced into cancer cell lines to conduct tumor cell transplantation experiments in nude mice. Notably, this system effectively reduced tumor sizes in mice injected with p53-deficient cells (G361, SCl-1 and HeLa) (Figure 6B). Based on these findings, we proposed that the dCas9-VP64-p53 switch could sense wild-type p53 and induce apoptosis in p53-deficient cancer cells.

## Discussion and Conclusion

The aptazyme switch is a widely used molecular tool for regulating gene function in biological research. The *p53* gene acts as a critical tumor suppressor, and its mutation often leads to tumorigenesis 37. Although some molecular tools and therapies have been developed for *p53* gene abnormalities 38, 39, few studies have focused on sensing endogenous p53 protein via the aptazyme switch, which in turn affects cellular function. Aptazyme switches normally require the addition of foreign molecules to be activated (such as tetracycline or theophylline), which may cause cytotoxicity and low permeability 40. If endogenous proteins could be sensed by the switch, these problems could be lessened to some extent. In previous studies, Win et al. developed a universal aptazyme switch control platform based on RNA. In this study, a series of aptamer ribozymes regulated by small molecules such as tetracycline or theophylline were constructed. However, cell activities are mainly controlled by proteins, and it is necessary to be able to sense macromolecules in synthetic genetic circuits. In our study, we attempted to construct an aptazyme switch that could sense endogenous p53 protein and distinguish normal cells from cancer cells.

Previously developed p53 sensors or mut-p53 inhibitors lack modularity. In this study, we first combined the p53 aptazyme switch with the Cre-loxP tool. The system specifically induces the deficiency of wild-type p53 protein and releases diphtheria toxin in tumor cells to kill the tumor cells. In wild-type p53 cells, only the EGFP gene is expressed without killing cells. The Cre-p53 aptazyme gene circuit indirectly reflects the level of endogenous p53 by fluorescence intensity and is targeted to kill tumor cells.

However, the expression of Cre may not effectively reflect the cutting efficiency for Loxp, and background leakage expression exists to some extent 41. Moreover, the introduction of multiple vectors into the cells led to low efficiency.

To resolve the above problem, we decided to use the switch in combination with dCas9-VP64 to activate endogenous p53 expression for tumor suppression. Numerous cancer cells show abnormalities in the *p53* gene (suppressed activity or heterozygous mutation). In the absence of wild-type p53 protein, the fusion protein dCas9-VP64 bound to the promoter region of the p53 gene and activated its expression under the guidance of p53 sgRNA, which induced tumor cell apoptosis. This design outperformed the Cre-p53 aptazyme by simplifying the tumor-targeted killing process using a single vector to simultaneously achieve sensing and killing. In addition, it avoided the expression of exogenous killing genes and eliminated the toxic effects of leaky DT. The tool was ineffective in wild-type p53 cells, but it was effective in activating the wild-type p53 allele in p53 heterozygous mutant or wild-type p53 suppressed cells. However, we also found that activating the functional mutant p53 gene may promote the growth of tumor cells (such as bladder cancer cell- T24). p53 functional mutations occupied an important position in the p53 mutation type, which was the next problem to be solved. The selective use of Cre-p53 aptazyme or CRISPR-p53 aptazyme tools in different situations would be a more rational choice.

In summary, this study not only developed a novel and efficient ribozyme switch for p53-specific recognition but also provided a modular strategy for aptazyme binding to cellular proteins. In addition, the p53 aptazyme successfully inhibited tumor growth through a combined application with other synthetic biological tools, providing a new perspective for cancer therapy.

## Supplementary Material

Supplementary figures and table.
