# Supplementary material for: Synthesis of RNA-based gene regulatory devices for redirecting cellular signaling events mediated by p53
Source: Theranostics. 2021 Mar 4;11(10):4688–98. doi: 10.7150/thno.55856 (PMC7978309; doi:10.7150/thno.55856)
Supplement: Supplementary file 1 — Supplementary figures and table. [file thnov11p4688s1.pdf]

Synthesis of RNA-based gene regulatory devices for redirecting cellular signaling events mediated by p53

Xinbo Huang et al.

Supplementary Information

Table S1

Name	cDNA sequence
p53 aptamer	GGGCGAATTCGGGTTGGATAGTAGGCGCATATGGCATCTTCGTGG<br>TTGTGTATTGCCCTTTAGTGAGGGTTAATT
p53 aptazyme	AAACAAACAAAGCTGTCACCGGATGTGCTTTCCGGTCTGATGAG<br>TCCGTCCTGGGGGCGAATTCGGGTTGGATAGTAGGCGCATATGGC<br>ATCTTCGTGGTTGTGTATTGCCCTTTAGTGAGGGTTAATTTCATAG<br>AGGACGAAACAGCAAAAAGAAAAATAAAAA
p53 sgRNA	GCATCCCGGATCAGATTTCG
hTERT promoter	GGCCCCTCCCTCGGGTTACCCCACAGCCTAGGCCGATTCGACCTC<br>TCTCCGCTGGGGCCCTCGCTGGCGTCCCTGCACCCTGGGAGCGC<br>GAGCGGCGCGCGGGCGGGGAAGCGCGGCCCAGACCCCCGGGTC<br>CGCCCGGAGCAGCTGCGCTGTCGGGGCCAGGCCGGGCTCCCAGT<br>GGATTCGCGGGCACAGACGCCCAGGACCGCGCTCCCCACGTGGC<br>GGAGGGACTGGGGACCCGGGCACCCGTCCTGCCCCTTCACCTTC<br>CGGCTCCGCCTCCTCCGCGCGGACCCCGCCCCGTCCCGACCCCTT<br>CCGGGTTTCCGGCCCAGCCCCTTCCGGGCCCTCCCAGCCCCTCCC<br>CTTCCTTTCCGGGGCCCCGCCCTCTCCTCGCGGCGCGAGTTTCCG<br>GCAGCGCTGCGTCCTGCTGCGCACGTGGGAAGCCCTGGCCCCGG<br>CCACCCCCGCG

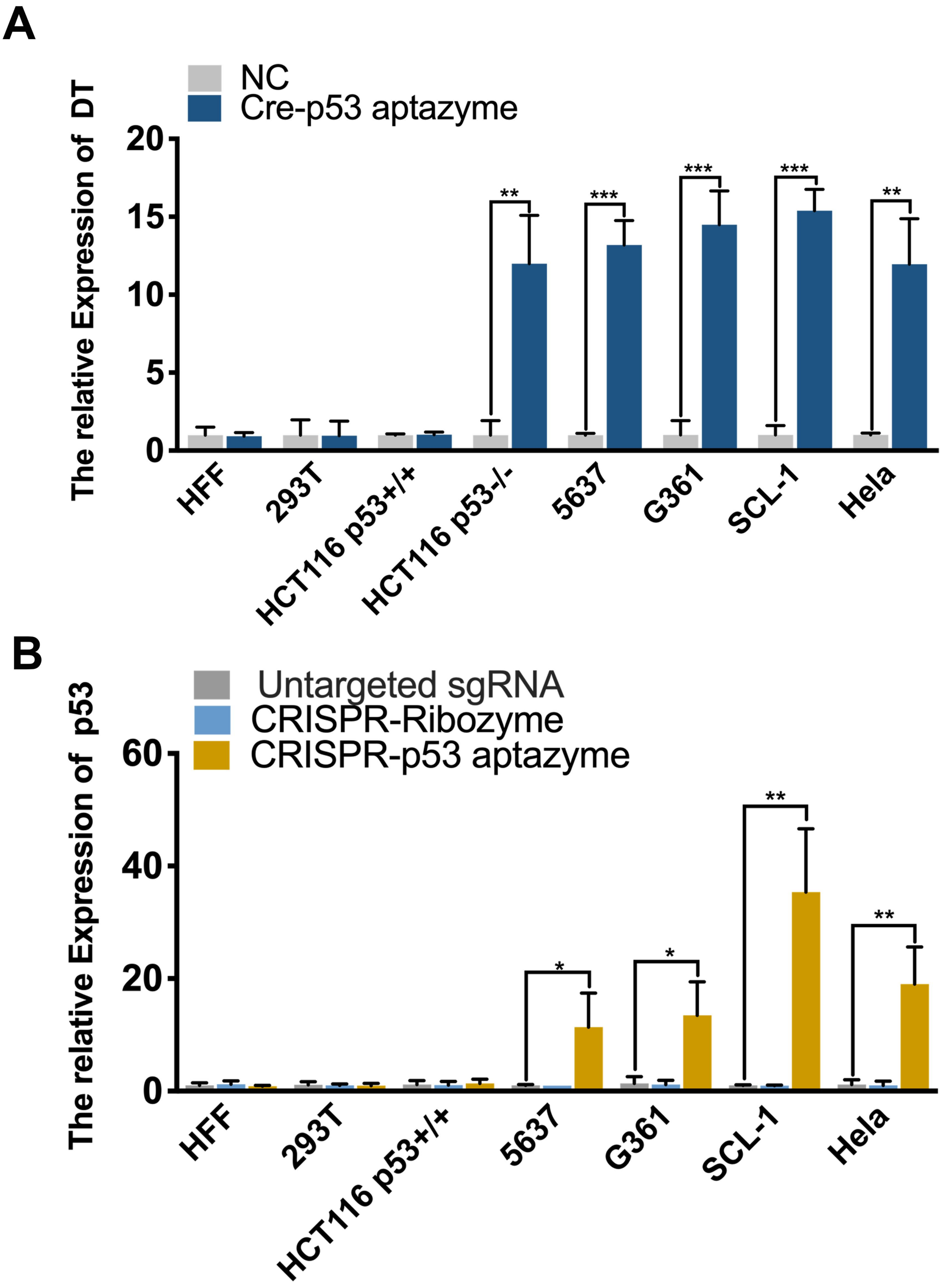

Figure S1. The expression of DT in Cre-aptazyme system and p53 in CRISPR-aptazyme system: (A) Quantitative real-time PCR representing the expression level of DT in Cre-p53 aptazyme group and negative control in wild-type p53 deficient cells (HCT116 p53-/-, 5637, G361, SCL-1 and Hela). **(B)** Quantitative real-time PCR representing the expression of p53 in CRISPR-p53 aptazyme group,

CRISPR-Ribozyme group and Untargeted sgRNA group in wild-type p53 deficient cells (5637, G361, SCL-1 and Hela). Data are presented as the means ± SD from at least three biological replicates. ($^{*}P < 0.05$, $^{**}P < 0.01$, $^{***}P < 0.001$)

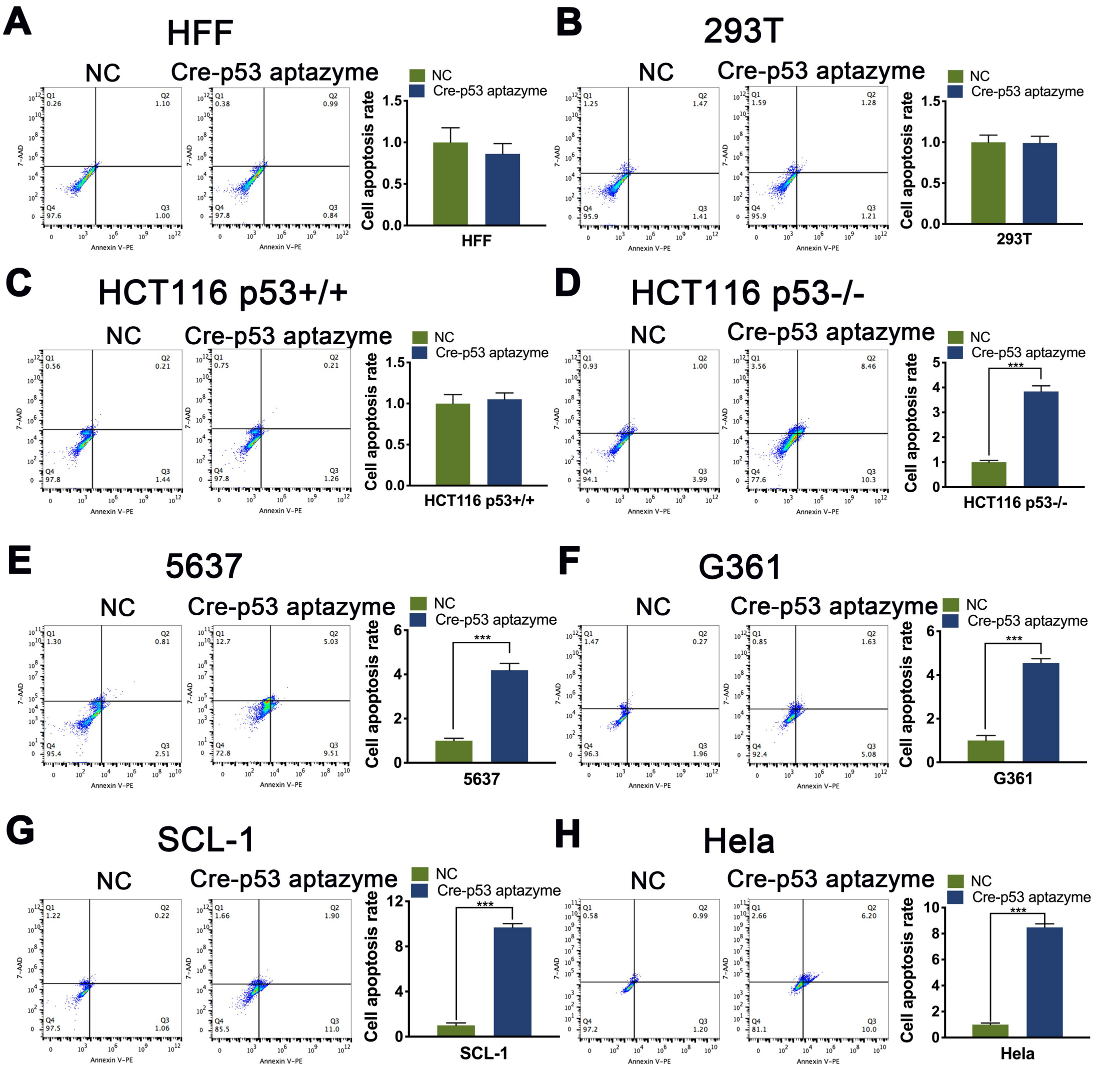

Figure S2. Cre-p53 aptazyme system promoted the apoptosis in wild-type p53 deficient cells: (A-H) Quantified results of the apoptosis between the NC group and the Cre-p53 aptazyme groups in different cells via flow cytometry. **(A-C)** In HFF cell, 293T cells, HCT116 p53+/+ (wild-type p53) cells respectively. **(D-H)** In HCT116 p53-/-, 5637, G361, SCL-1 and Hela (wild-type p53 deficient) cells respectively. Data are presented as the means ± SD from at least three biological replicates. ($^{*}P < 0.05$, $^{**}P < 0.01$, $^{***}P < 0.001$)

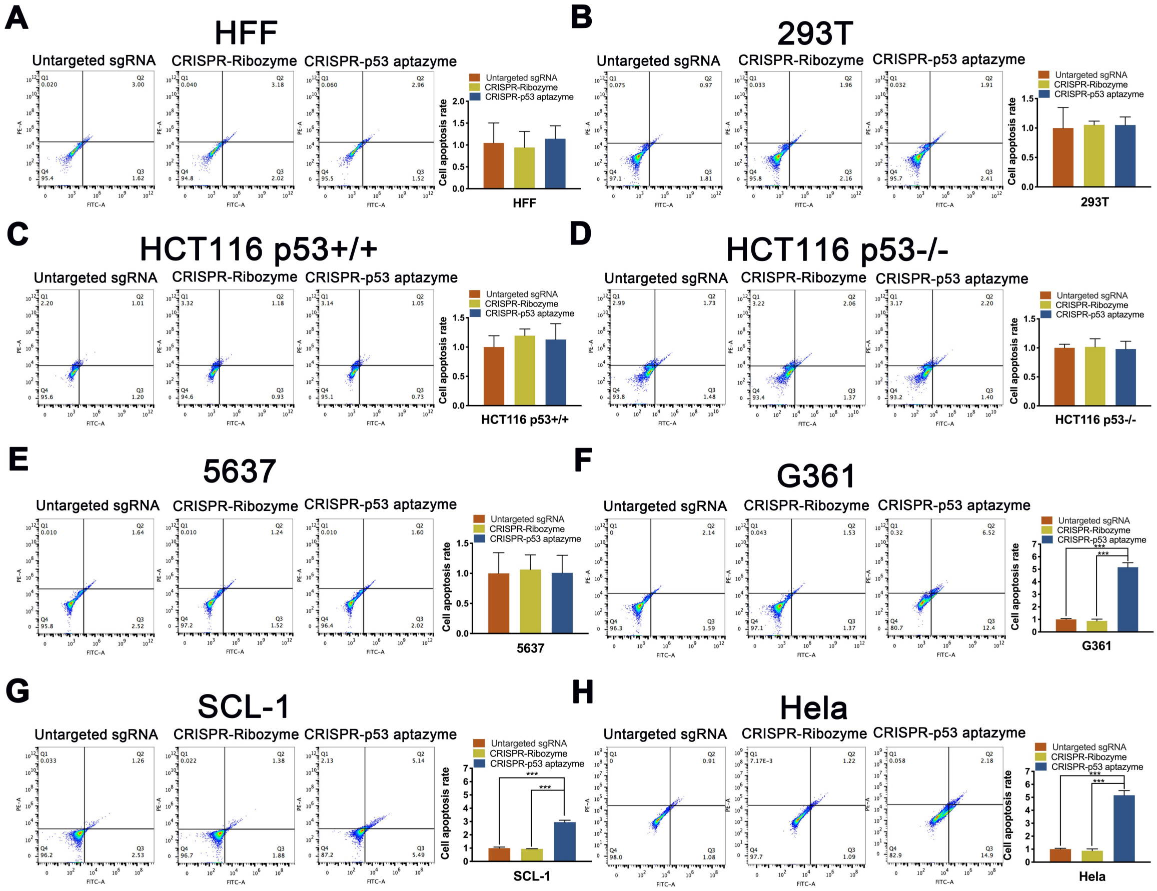

Figure S3. CRISPR-p53 aptazyme system promoted the apoptosis in some wild-type p53 deficient cells: (A-H) Quantified results of the apoptosis between the NC group and the Cre-p53 aptazyme groups in different cells via flow cytometry. **(A-C)** In HFF, 293T and HCT116 p53+/+ (wild-type p53) cells respectively. **(D-H)** In HCT116 p53-/-, 5637, G361， SCL-1 and Hela (wild-type p53 deficient) cells respectively. Data are presented as the means ± SD from at least three biological replicates. ($^{*}P < 0.05$, $^{**}P < 0.01$, $^{***}P < 0.001$)

Figure S4. Maps of plasmids used in this study.

A	psi-SV40 promoter-hRluc-p53 aptazyme-HSV TK promoter-hluc
B	pZDonor-hTERT-Cre- p53 aptazyme-rb terminator
C	pZDonor-hEF1a-loxp71-EGFP-loxp66-Diphtheria Toxin
D	pZDonor-U6-p53 sgRNA-hTERT promoter-NLS-dcas9-NLS-VP64-p53 aptazyme
E	pZDonor-U6-p53 sgRNA-hTERT promoter-NLS-dcas9-NLS-VP64-Ribozyme
F	pZDonor-U6- untargeted sgRNA -hTERT promoter-NLS-dcas9-NLS-VP64- p53 aptazyme

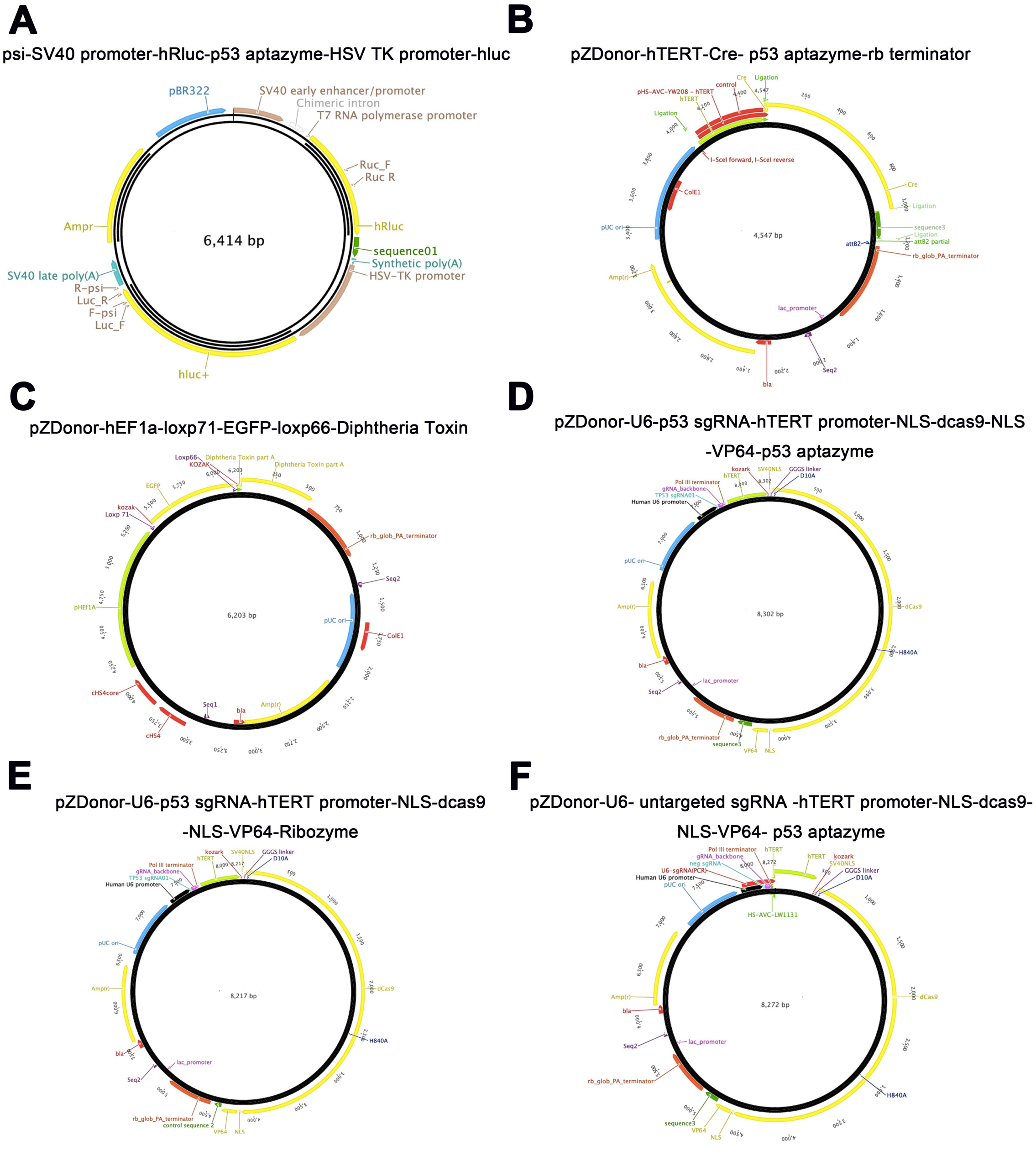
A
psi-SV40 promoter-hRluc-p53 aptazyme-HSV TK promoter-hluc
pBR322
SV40 early enhancer/promoter
Chimeric intron
T7 RNA polymerase promoter
Ruc_F
Ruc R
hRluc
sequence01
Synthetic poly(A)
HSV-TK promoter
hluc+
Luc_F
F-psi
Luc_R
R-psi
SV40 late poly(A)
Ampr
6,414 bp
B
pZDonor-hTERT-Cre- p53 aptazyme-rb terminator
pHS-AVC-YW208 - hTERT
hTERT
control
Cre
Ligation
I-SceI forward, I-SceI reverse
ColE1
pUC ori
Amp(r)
Cre
Ligation
sequence3
Ligation
attB2 partial
attB2
rb_glob_PA_terminator
lac_promoter
Seq2
bla
4,547 bp
C
pZDonor-hEF1a-loxp71-EGFP-loxp66-Diphtheria Toxin
Loxp66
KOZAK
Diphtheria Toxin part A
EGFP
kozak
Loxp 71
pHEf1A
cHS4core
cHS4
Seq1
bla
Amp(r)
pUC ori
ColE1
Seq2
rb_glob_PA_terminator
6,203 bp
D
pZDonor-U6-p53 sgRNA-hTERT promoter-NLS-dcas9-NLS
-VP64-p53 aptazyme
Pol III terminator
gRNA_backbone
TP53 sgRNA01
Human U6 promoter
hTERT
kozak
SV40NLS
GGGS linker
D10A
dCas9
H840A
pUC ori
Amp(r)
bla
Seq2
lac_promoter
rb_glob_PA_terminator
sequence3
VP64
NLS
8,302 bp
E
pZDonor-U6-p53 sgRNA-hTERT promoter-NLS-dcas9
-NLS-VP64-Ribozyme
Pol III terminator
gRNA_backbone
TP53 sgRNA01
Human U6 promoter
hTERT
kozak
SV40NLS
GGGS linker
D10A
dCas9
H840A
pUC ori
Amp(r)
bla
Seq2
lac_promoter
rb_glob_PA_terminator
control sequence 2
VP64
NLS
8,217 bp
F
pZDonor-U6- untargeted sgRNA -hTERT promoter-NLS-dcas9-
NLS-VP64- p53 aptazyme
Pol III terminator
gRNA_backbone
neg sgRNA
U6-sgRNA(PCR)
Human U6 promoter
pUC ori
hTERT
kozak
SV40NLS
GGGS linker
D10A
HS-AVC-LW1131
dCas9
H840A
Amp(r)
bla
Seq2
lac_promoter
rb_glob_PA_terminator
sequence3
VP64
NLS
8,272 bp

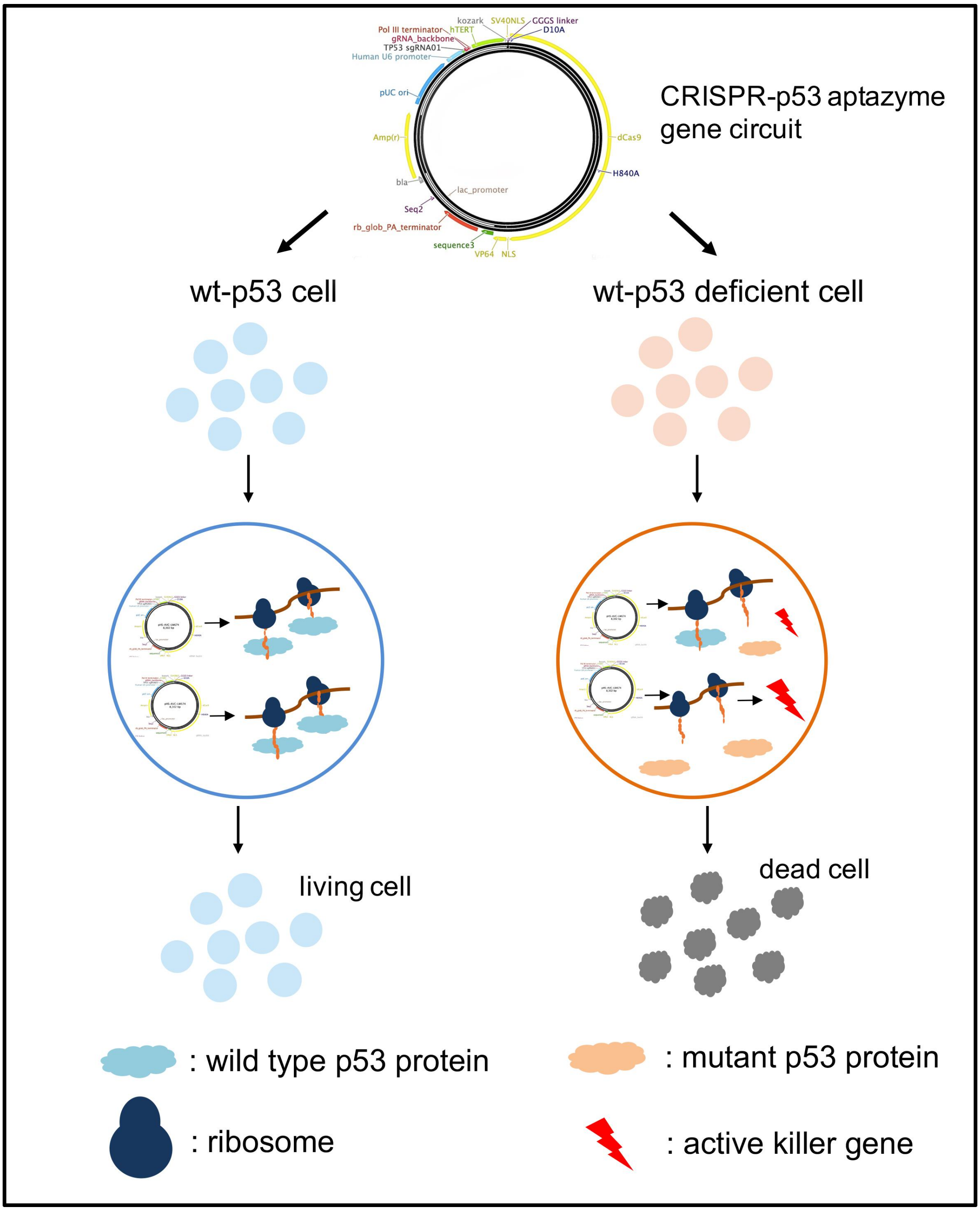

Figure S5. The operating mode of CRISPR-p53 aptazyme system in cells: A functional model of the CRISPR-p53 aptazyme system inside the wild-type p53 and mutant p53 cells was presented. In the absence of wild-type p53, the system initiated targeted killing effect.

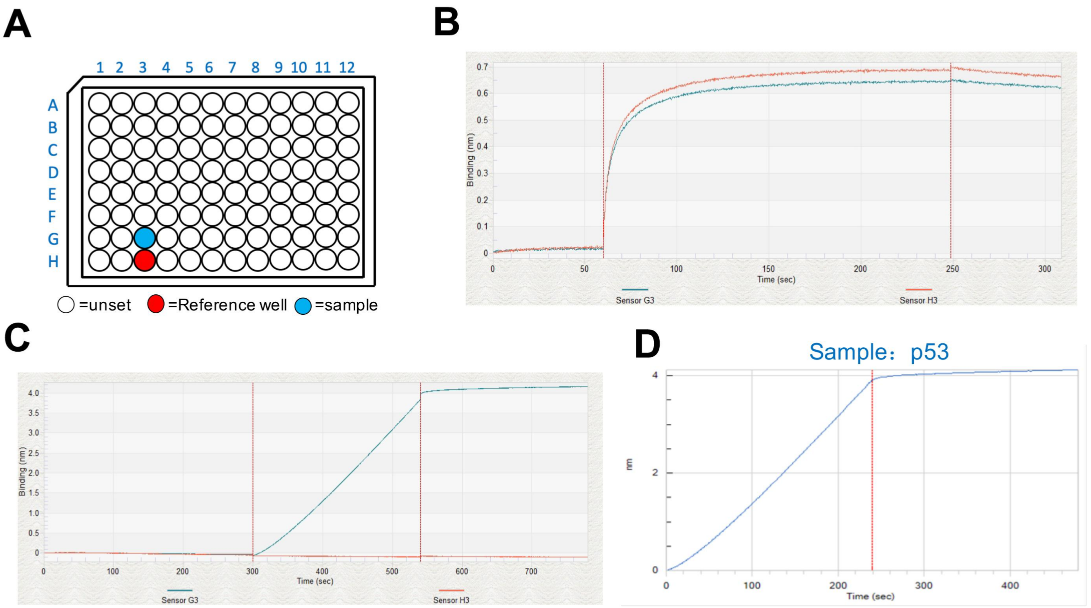

Figure S6. Detection of the binding effect between p53 protein and the p53 aptamer: (A) Schematic diagram indicating the position of reference well and sample well. The blue represents the sample to be tested, and the red represents the control sample, the colour scheme is consistent with the curves in the following pictures. **(B)** The p53 aptamer sequences were successfully immobilized on the SA chips of the reference and sample wells. **(C, D)** The p53 protein was successfully combined with the p53 aptamer RNA sequence (blue curve).

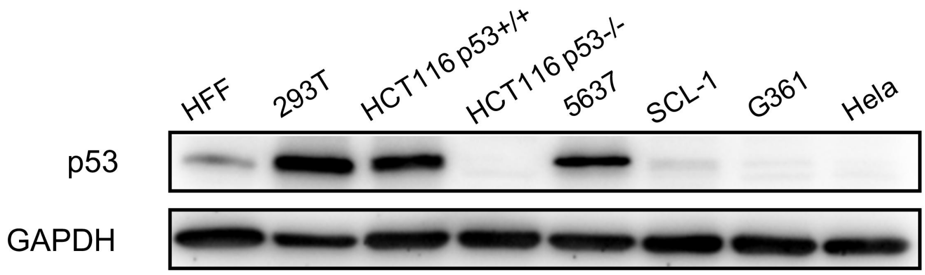

Figure S7. Expression of total p53 protein in various cell lines.

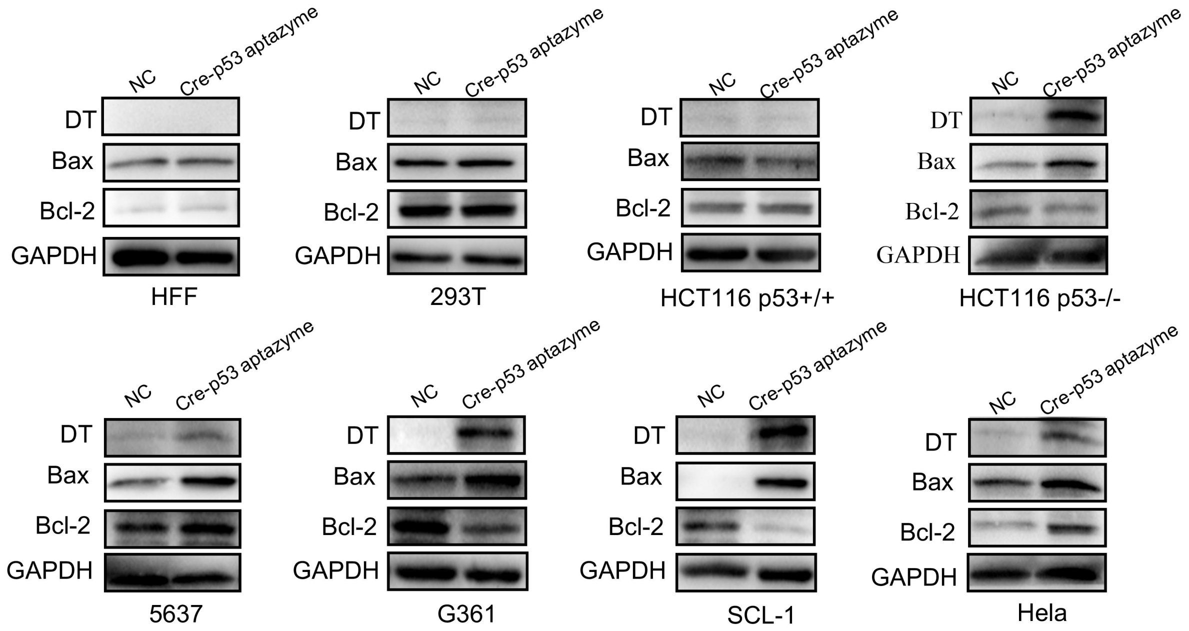

Figure S8. The expression of DT protein and apoptosis-related protein in Cre-p53 aptazyme gene circuit: There was almost no expression of DT protein in HFF, 293T and HCT116 p53+/+ cells, but its expression was significantly increased in the Cre- p53 aptazyme group of HCT116 p53+/+, 5637, G361, SCl-1 and Hela cells. Correspondingly, the expression of apoptosis-related proteins Bax/Bcl-2 also increased.

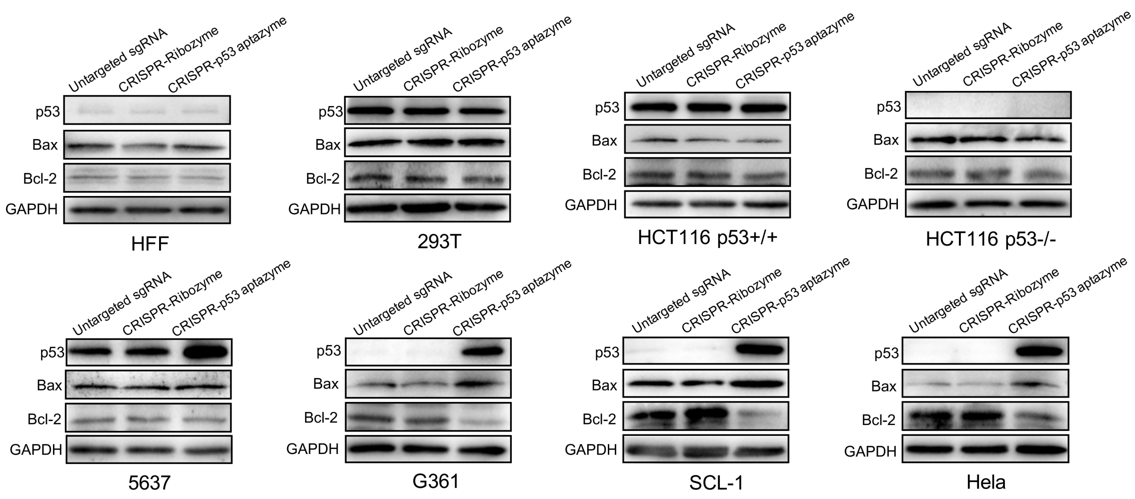

Figure S9. The expression of p53 protein and apoptosis-related protein in CRISPR-p53 aptazyme gene circuit: In HFF, 293T and HCT116 p53+/+ cells, the expression of p53 protein in the CRISPR-p53 aptazyme group and the control groups were not significantly activated. In HCT116 p53-/- cells, p53 protein was not expressed. In 5637, G361, SCL-1 and Hela cells, the expression of p53 protein in the CRISPR-p53 aptazyme group was significantly increased. The expression of apoptosis-related proteins Bax/Bcl-2 increased in G361, SCL-1 and Hela cells.
